# Supplementary material for: Establishing Normative Values to Determine the Prevalence of Biochemical Hyperandrogenism in Premenopausal Women of Different Ethnicities from Eastern Siberia
Source: Diagnostics (Basel). 2022 Dec 22;13(1):33. doi: 10.3390/diagnostics13010033 (PMC9818891; doi:10.3390/diagnostics13010033)
Supplement: Supplementary file 1 [file diagnostics-13-00033-s001.zip › diagnostics-1999278-supplementary.pdf]

Table S1. Socio-demographic characteristics of healthy controls by ethnicity.

**Parameters**	**Caucasians n = 88 (1)**	**Asians n = 42 (2)**	**Mixed n = 13 (3)**	***p*-Value**
Age, years,<br>Mean±SD<br>Median (LQ;UQ)	34.2±6.13<br>35.0<br>(30.0;39.0)	36.2±5.4<br>38.0<br>(32.0;40.0)	33.0±6.2<br>34.0 (32.0;37.0)	$p_U = 0.66^{1-2}$<br>$p_U = 0.75^{1-3}$<br>$p_U = 0.97^{2-3}$
Marital status, n/N (%)				
Single	17/88 (19.3%)	11/42 (26.1%)	3/13 (2.10%)	$p_{\chi^2}^{1.2.3}=0.62$
Married	49/88 (55.6%)	21/42 (50.0 %)	10/13 (76.9%)	$p_{\chi^2}^{1.2.3}=0.23$
Living with another	12/88 (13.6%)	3/42 (7.14%)	0/13 (0.00%)	$p_{\chi^2}^{1.2.3}=0.26$
Separated	0/88 (0.00%)	0/42 (0.00%)	0/13 (0.00%)	
Divorced	8/88 (9.09%)	5/42 (11.9%)	0/13 (0.00%)	$p_{\chi^2}^{1.2.3}=0.43$
Widowed	1/88 (1.14%)	1/42 (2.38%)	0/13 (0.00%)	$p_{\chi^2}^{1.2.3}=0.78$
Would rather not say	1/88 (1.14%)	1/42 (2.38%)	0/13 (0.00%)	$p_{\chi^2}^{1.2.3}=0.78$
Occupation, n/N (%)				
Legislators. senior officials. and managers	3/88 (3.45%)	1/42 (2.44%)	0/13 (0.00%)	$p_{\chi^2}^{1.2.3}=0.75$
Professionals	35/88 (40.2%)	25/42 (60.9%)	5/13 (38.4%)	$p_{\chi^2}^{1.2.3}=0.06$<br>$p_{\chi^2}=0.02^{1-2}$<br>$p_{\chi^2 Fisher}=0.57^{1-3}$<br>$p_{\chi^2 Fisher}=0.13^{2-3}$
Technicians and associate professionals	16/88 (18.3%)	5/42 (12.2%)	5/13 (38.4%)	$p_{\chi^2}^{1.2.3}=0.10$
Office clerks	10/88 (11.4%)	6/42 (14.6%)	1/13 (7.6%)	$p_{\chi^2}^{1.2.3}=0.78$
Service workers. and shop and market sales	6/88 (6.90%)	3/42 (7.32%)	0/13 (0.00%)	$p_{\chi^2}^{1.2.3}=0.60$
Skilled agricultural and fishery workers	2/88 (2.30%)	0/42 (0.00 %)	0/13 (0.00%)	$p_{\chi^2}^{1.2.3}=0.52$
Craft and related trades workers	9/88 (10.3%)	1/42 (2.44%)	1/13 (7.69%)	$p_{\chi^2}^{1.2.3}=0.27$
Plant and machine operators and assemblers	2/ 88 (2.30%)	0/42 (0.00%)	0/13 (0.00%)	$p_{\chi^2}^{1.2.3}=0.72$

Elementary occupations	4/ 88 (4.60%)	0/42 (0.00%)	0/13 (0.00%)	$p_{\chi2}^{1.2.3}$=0.26
Armed forces	0/88 (0.00%)	0/42 (0.00%)	1/13 (7.69%)	
Missing data on occupation	1/88 (1.14%)	1/42 (2.44%)	0/13 (0.00%)	
		Education, n/N(%)		
Doctoral degree	8/ 88 (8.24%)	3/42 (7.14 %)	5/13 (38.46%)	$p_{\chi2}^{1.2.3}$=0.003<br>$p_{\chi2Fisher}$=0.49^{1-2}<br>$p_{\chi2Fisher}$=0.01^{1-3}<br>$p_{\chi2Fisher}$=0.01^{2-3}
Master's degree	51/88 (58.8%)	36/42 (85.7%)	7/13 (53.8%)	$p_{\chi2}^{1.2.3}$=0.006<br>$p_{\chi2}$= 0.002^{1-2}<br>$p_{\chi2\ Yates}$=0.78^{1-3}<br>$p_{\chi2\ Yates}$=0.04^{2-3}
Bachelor's degree	3/88 (3.53 %)	0/42 (0.00 %)	0/13 (0.00 %)	$p_{\chi2}^{1.2.3}$=0.37
Some college	17/88 (18.8%)	3/42 (7.14%)	1/13 (7.69%)	$p_{\chi2}^{1.2.3}$=0.16
High school or equivalent	1/88 (1.18%)	0/42 (0.00%)	0/13 (0.00%)	$p_{\chi2}^{1.2.3}$=0.72
Incomplete high school	5/88 (5.88%)	0/42 (0.00%)	0/13 (0.00%)	$p_{\chi2}^{1.2.3}$=0.18
Middle school only	2/88 (2.35%)	0/42 (0.00%)	0/13 (0.00%)	$p_{\chi2}^{1.2.3}$=0.51
No degree	1/88 (1.18%)	0/42 (0.00%)	0/13 (0.00 %)	$p_{\chi2}^{1.2.3}$=0.72

$\chi2$ - Pearson Chi-square and Fisher exact one-tailed tests. U - Mann-Whitney U Test

Table S2. Menstrual and reproductive history of healthy controls by ethnicity.

**Parameters**	**Caucasians n = 88 (1)**	**Asians n = 42 (2)**	**Mixed n = 13 (3)**	***p*-value***
	Mean±SD<br>Median (LQ;UQ)			
Age at menarche, years	13.1±1.21<br>13.0<br>(12.0;14.0)	13.5±1.25<br>13.00<br>(13.0;14.0)	13.2±1.09<br>13.0<br>(13.0;14.0)	$p^{1,2,3}$=0.33
Min length of menstrual cycle, days	26.3±2.24<br>26.0<br>(25.0;28.0)	26.7±2.06<br>27.0 (25.0;28.0)	26.2±2.49<br>26.0 (25.0;28.0)	$p^{1,2,3}$=0.78
Max length of menstrual cycle, days	29.3±2.23<br>30.0<br>(28.0;30.0)	29.9±3.94<br>30.0<br>(28.0;30.0)	29.6±2.57<br>30.0 (27.0;32.0)	$p^{1,2,3}$=0.95
Number of pregnancies	2.3±2.2<br>2.0<br>(1.0; 3.0)	2.3±1.8<br>2.0<br>(1.0; 3.0)	2.6±1.4<br>2.0<br>(2.0; 3.0)	$p^{1,2,3}$ =0.52
Live births	1.7±0.7<br>2.0<br>(1.0; 2.0)	1.9±0.9<br>2.0<br>(1.0; 3.0)	1.8±0.6<br>2.0<br>(1.0; 2.0)	$p^{1,2,3}$ =0.29
Still birth	0.05±0.2<br>0.00	0.05±0.3<br>0.00	0.0±0.0<br>0.00	$p^{1,2,3}$ =0.51

	(0.00; 0.00)	(0.00; 0.00)	(0.00; 0.00)	
Spontaneous abortions	0.1±0.4<br>0.00<br>(0.00; 0.00)	0.2±0.5<br>0.00<br>(0.00; 0.00)	0.1±0.3<br>0.00<br>(0.00; 0.00)	$p^{1,2,3}$=0.63
Extrauterine pregnancy	0.04±0.2<br>0.00<br>(0.00; 0.00)	0.05±0.3<br>0.00<br>(0.00; 0.00)	0.1±0.3<br>0.00<br>(0.00; 0.00)	$p^{1,2,3}$=0.70
Missed abortion	0.1±0.4<br>0.00<br>(0.00; 0.00)	0.0±0.0<br>0.00<br>(0.00; 0.00)	0.2±0.6<br>0.00<br>(0.00; 0.00)	$p^{1,2,3}$=0.28
Medical abortions	0.8±1.5<br>0.00<br>(0.00; 1.00)	0.4±0.9<br>0.00<br>(0.00; 0.00)	0.9±1.1<br>1.00<br>(0.00; 1.00)	$p^{1,2,3}$=0.14

*Kruskal-Wallis ANOVA by Ranks.

Table S3. Anthropometry, vital signs and pelvic U/S parameters of healthy controls by ethnicity.

**Anthropometry And Vital Signs Parameters**	**Caucasians n = 88**	**Asians n = 42**	**Mixed n = 13**	***p*-Value***
	Mean±SD Median (LQ;UQ)			
Weight, kg	65.8±10.4<br>65.5<br>(57.1;74.3)	61.2±7.81<br>61.1 (55.3;66.0)	60.7±6.38<br>62.7<br>(54.2;65.0)	$p_{1\text{-}2}$=0,01<br>$p_{1\text{-}3}$=0,08<br>$p_{2\text{-}3}$=0,96
Height,cm	162±5.24<br>163<br>(158;167)	161±4.48<br>160<br>(158;165)	162±5.03<br>163<br>(158;165)	$p_{1\text{-}2}$=0,17<br>$p_{1\text{-}3}$=0,89<br>$p_{2\text{-}3}$=0,46
WC,cm	24.8±3.20<br>24.9<br>(22.1;27.8)	23.5±3.20<br>23.3<br>(20.8;25.7)	23.0±2.82<br>23.6<br>(20.4;25.1)	$p_{1\text{-}2}$=0,03<br>$p_{1\text{-}3}$=0,06<br>$p_{2\text{-}3}$=0,64
BMI,kg/m^2	75.4±8.9<br>76.5<br>(68.5;82.0)	75.4±8.2<br>75.5<br>(68.0;81.0)	74.3±8.6<br>72.0<br>(69.0;78.0)	$p_{1\text{-}2}$=0,83<br>$p_{1\text{-}3}$=0,53<br>$p_{2\text{-}3}$=0,62
Systolic blood pressure, mm Hg	118±9.60<br>117<br>(112;123)	117±11.13<br>117<br>(110;127)	115±9.05<br>117<br>(109;121)	$p_{1\text{-}2}$=0.78<br>$p_{1\text{-}3}$=0.428<br>$p_{2\text{-}3}$=0.677
Diastolic blood pressure, mm Hg	74.6±7.18<br>74.0<br>(69.5;79.0)	75.1±8.40<br>75.5<br>(69.0;82.0)	76.3±6.12<br>77.0 (71.0;82.0)	$p_{1\text{-}2}$=0.746<br>$p_{1\text{-}3}$=0.480<br>$p_{2\text{-}3}$=0.691
mFG score	0.43±0.72<br>0.00<br>(0.00;1.00)	0.21±0.47<br>0.00<br>(0.00;0.00)	0.31±0.63<br>0.00<br>(0.00;0.00)	$p_{1\text{-}2}$=0.139<br>$p_{1\text{-}3}$=0.596<br>$p_{2\text{-}3}$=0.711
Pelvic U/S	*Mean±SD Median (LQ;UQ)*			
AFC, right ovary	6.56±2.19<br>6.00<br>(5.00;8.00)	5.56±1.84<br>5.00 (4.00;7.00)	5.08±1.44<br>5.00 (4.00;6.00)	$p_{1\text{-}2}$=0.005<br>$p_{1\text{-}3}$=0.008<br>$p_{2\text{-}3}$=0.53
AFC, left ovary	6.44±2.39<br>6.00<br>(5.00;8.00)	5.65 ±2.05<br>5.00 (4.00;7.00)	5.39±1.81<br>6.00 (4.00;6.00)	$p_{1\text{-}2}$=0.07<br>$p_{1\text{-}3}$=0.17<br>$p_{2\text{-}3}$=0.87
Volume, right ovary, cm^3	7.33±6.69	6.29±2.32	5.14±1.86	$p_{1\text{-}2}$=0,48

	6.09 (5.11;7.77)	6.13 (4.74;7.96)	5.39 (3.43;6.50)	p_{1-3}=0,04 p_{2-3}=0,15
Volume, left ovary, cm³	6.25±1.96	5.40±1.96	5.68±1.85	p_{1-2}=0,01
	6.17 (4.83;7.29)	5.24 (4.05;6.45)	5.33 (4.57;6.92)	p_{1-3}=0,37 p_{2-3}=0,52

WC- waist circumference, BMI- body mass index, mFG score- modified Ferriman-Gallwey score, U/S-ultrasound, AFC-antral follicle count, * Mann-Whitney U Test.

Table S4. Androgen profile of healthy controls by age.

Parameters	<35 Years n = 64	≥35 Years n = 79	*p-Value**
	Mean ± SD Median *(LQ;UQ)*		
TT, ng/dl	26.1±14.0 25.8 (17.3, 34.2)	24.2±15.5 22.0 (13.3, 33.7)	0.22
FAI	1.56±1.41 1.22 (0.60, 2.14)	1.48±1.70 1.20 (0.59, 1.85)	0.71
DHEAS, µg/dl	171±76.9 165 (118, 210)	149±65.9 145 (93.2, 185)	0.08

Abbreviations: TT-total testosterone, FAI—free androgen index, DHEAS- dehydroepiandrosterone sulfate. * Mann-Whitney U Test.

Table S5. The UNLs as defined by the 98th percentiles in healthy controls by age.

Parameters	<35 Years n = 64	≥35 Years n = 79
	**98th Percentile (95%CI)**	
TT, ng/dl	55.0 (44.2, 73.0)	70.3 (44.1, 78.0)
FAI	6.29 (3.04,7.32)	4.52 (2.54, 14.0)
DHEAS, µg/dl	366 (284, 374)	328 (234, 355)

Abbreviations: TT-total testosterone, FAI—free androgen index, DHEAS- dehydroepiandrosterone sulfate.

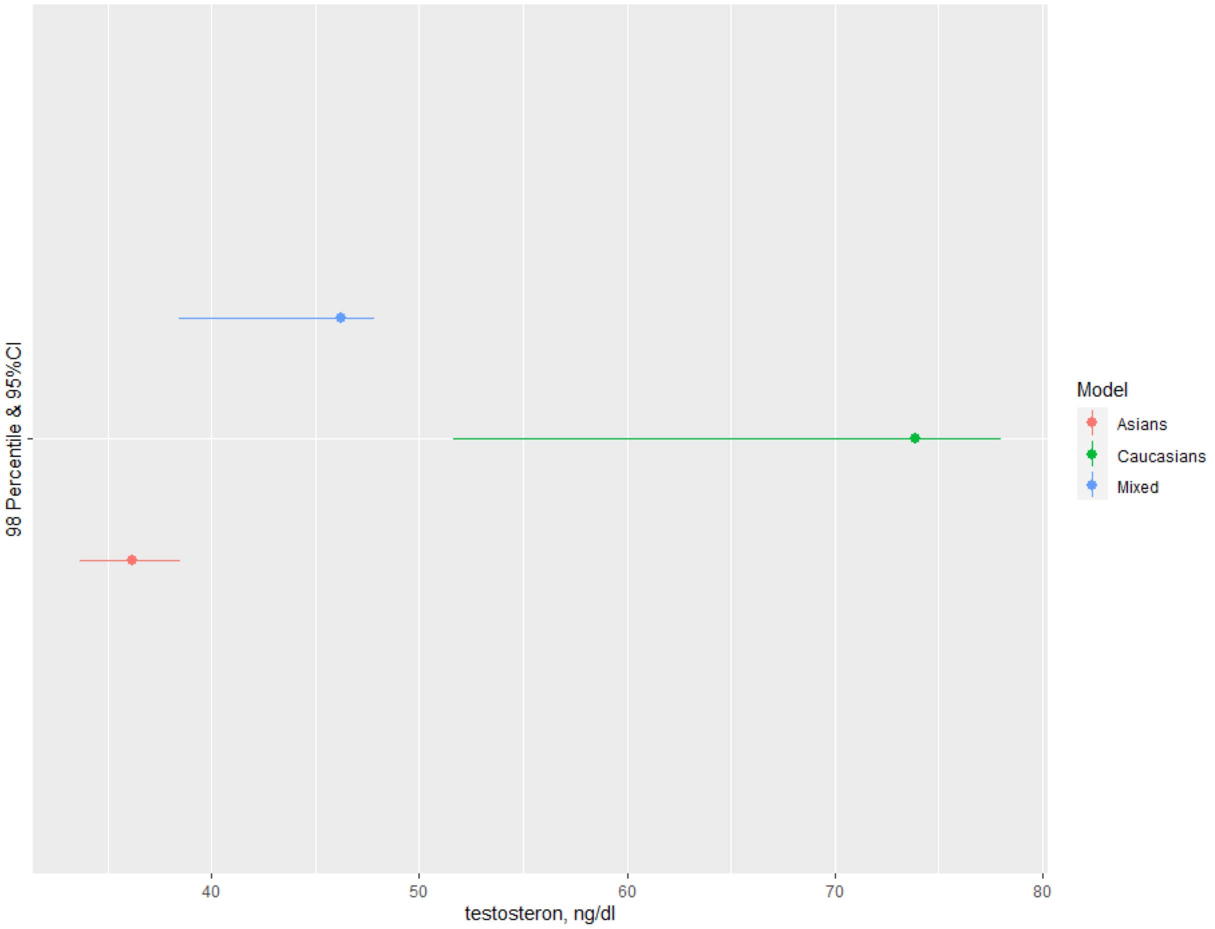

Figure S1. UNLs for the total testosterone (TT) by ethnicity: the analysis of the 95% CIs overlapping.

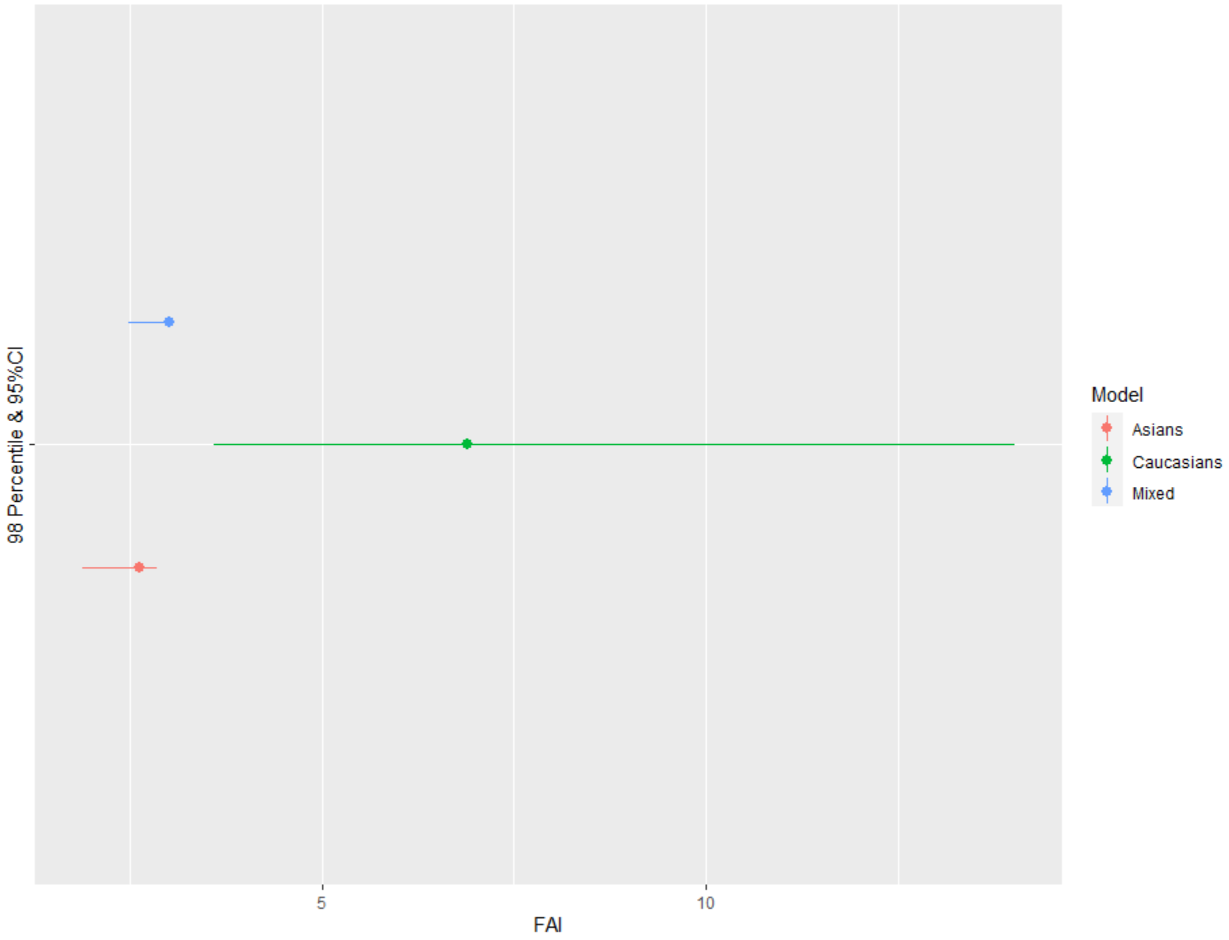

Figure S2. UNLs for free androgen index (FAI) by ethnicity: the analysis of the 95% CIs overlapping.

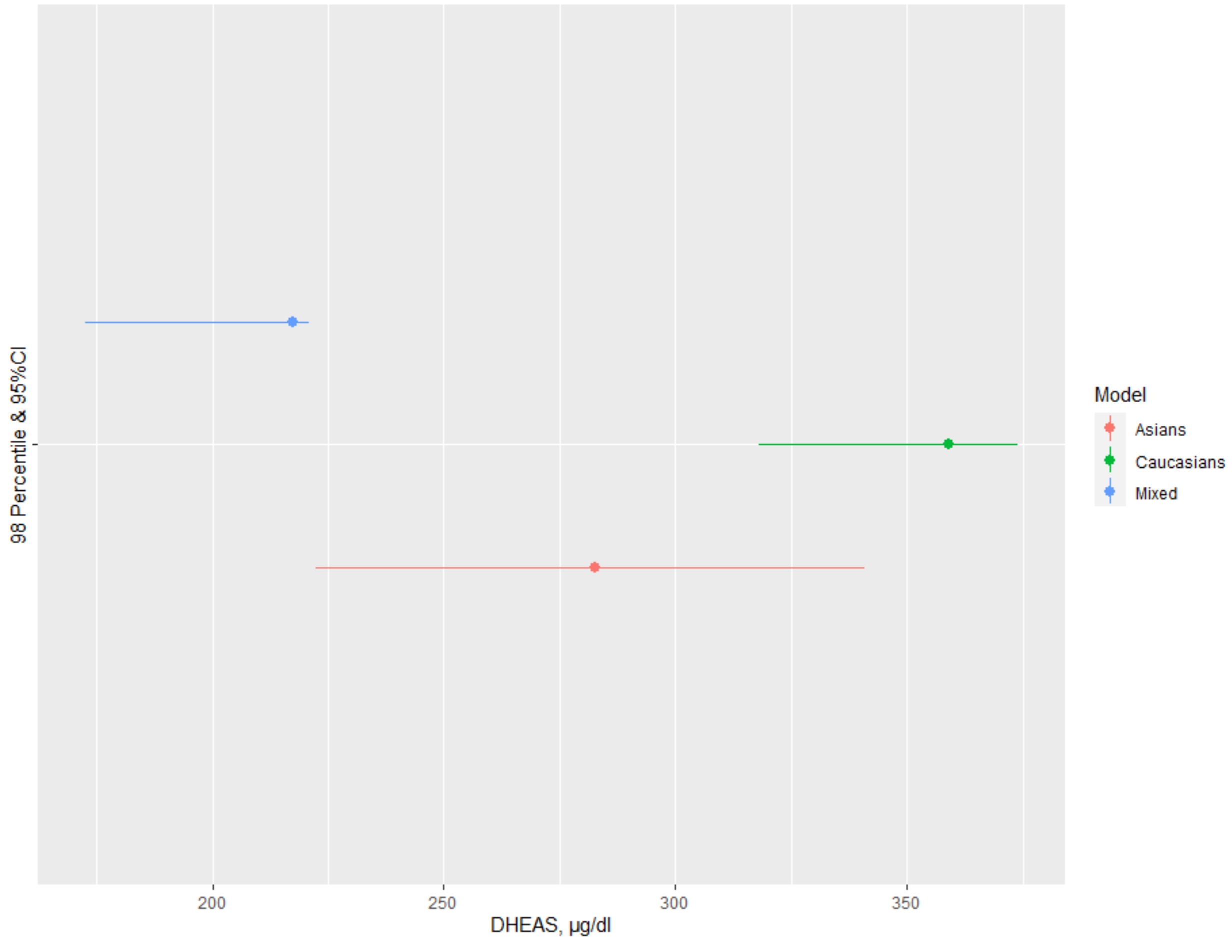

Figure S3. UNLs for DHEAS by ethnicity: the analysis of the 95% CIs overlapping.
